# Cost-effectiveness of different screening strategies for Down syndrome: a real-world analysis in 140,472 women

**DOI:** 10.3389/fpubh.2025.1535381

**Published:** 2025-05-09

**Authors:** Jing Liu, Shunyao Wang, Shihao Zhou, Di Ma, Lanping Hu, Hui Huang, Siyuan Linpeng, Jiawei Lin, Liu Hong, Qinmei Fu, Huanhuan Peng, Lijian Zhao, Nan Wang, Jun He

**Affiliations:** ^1^Hunan Provincial Key Laboratory of Regional Hereditary Birth Defects Prevention and Control, Changsha Hospital for Maternal & Child Health Care Affiliated to Hunan Normal University, Changsha, China; ^2^BGI Genomics, Shenzhen, China; ^3^MGI Tech, Shenzhen, China; ^4^Hebei Industrial Technology Research Institute of Genomics in Maternal & Child Health, Shijiazhuang BGI Genomics Co, Ltd, Shijiazhuang, Hebei, China; ^5^Medical Technology College of Hebei Medical University, Shijiazhuang, China; ^6^School of Public Administration, Hunan University, Changsha, Hunan, China

**Keywords:** prenatal diagnosis, health economics, health policy, non-invasive prenatal testing, Down syndrome

## Abstract

**Background:**

Recent advancements in high-throughput sequencing have validated the accuracy, safety, and effectiveness of non-invasive prenatal testing (NIPT) for Down syndrome (DS).

**Methods:**

This study aims to assess the effectiveness and economic implications of NIPT versus second-trimester serum screening (STSS) for DS and the different screening strategies through retrospectively analyzing data from 140,472 pregnant women who completed both NIPT and STSS (provided for free by local public welfare programs) between March 1, 2018 and December 31, 2020. Data were categorized into eight groups based on different screening strategies.

**Results:**

The sensitivity, specificity, and positive predictive value of NIPT for detecting trisomy 21 were significantly higher compared with those of STSS. The universal NIPT screening strategy demonstrated the best effectiveness, detecting 163 DS cases with the highest net benefit and a cost-effectiveness ratio of 1:9.53. The STSS and NIPT combined screening strategy detected 128 DS cases with the lowest cost-effectiveness at RMB 341,800. The incremental cost-effectiveness ratio of the universal NIPT screening strategy was RMB 1,186,200, significantly lower than the socioeconomic burden associated with a DS case.

**Conclusion:**

NIPT demonstrated significantly superior testing performance compared to STSS. At a unit cost of RMB 600, the universal NIPT screening strategy is the most effective and holds substantial health economic value.

## 1 Introduction

Maternal serum testing is the primary method for screening fetal chromosomal abnormalities in China. However, it has a relatively high false-positive rate and low accuracy (1). Recent advancements in high-throughput sequencing have demonstrated the accuracy, safety, and effectiveness of non-invasive prenatal testing (NIPT) for detecting fetal chromosomal aneuploidies (2–4). NIPT is favored for its technical advantages and is recommended by guidelines in many countries for prenatal screening (5–8). In the Netherlands and Belgium, NIPT is fully reimbursed and offered as a first-tier screening test nationwide (9, 10). However, current Chinese guidelines recommend using NIPT as a second-tier screening following the identification of high-risk factors through serum screening or in specific conditions, such as advanced maternal age or a family history of chromosomal abnormalities (11).

NIPT has superior sensitivity, specificity, and positive predictive value compared with serum screening (4, 12, 13). The health economic value of different screening strategies, including NIPT, serum screening, and contingent screening, has also been investigated in recent comparative analyses (14, 15). Studies from Belgium and the Netherlands suggest that NIPT, when used in conjunction with serum screening, offers a cost-effectiveness advantage (16, 17). Furthermore, results from a Chinese study indicate that NIPT as a contingent screening strategy has optimal health economic benefits (18). Due to its sensitivity, NIPT may hold higher health economic value considering the societal and economic burdens associated with Down syndrome (DS) (19). However, previous studies have often been limited by small sample sizes and simulated models. The high cost of NIPT remains a critical factor in decision-making (20). The Changsha municipal public welfare program’s reduction in NIPT costs provides an opportunity to re-evaluate and compare screening strategies using real-world data from this program, offering valuable insights into the health economic value of NIPT and serum screening.

This study retrospectively analyzed clinical data from 140,472 pregnant women who underwent both NIPT and second-trimester serum screening (STSS) between March 1, 2018, and December 31, 2020. It represents the first large-scale real-world study in China evaluating the performance and health economic value of NIPT and various screening strategies. This study analyzed outcomes from a healthcare system perspective, including medical costs in the calculations, allowing for a more comprehensive comparison of the health economic outcomes of different strategies. Thus, it provides decision-makers with crucial insights into the economic value and efficiency of various screening strategies within real-world healthcare settings. Additionally, the findings in this study offer crucial real-world data for foundational research on birth defect prevention in China and provide a scientific basis for evidence-based decision-making in clinical practice and policy development.

## 2 Materials and methods

### 2.1 Patient and public involvement

Patients and public were not involved in the design and conduct of the study.

### 2.2 Data source

**Figure 1:**
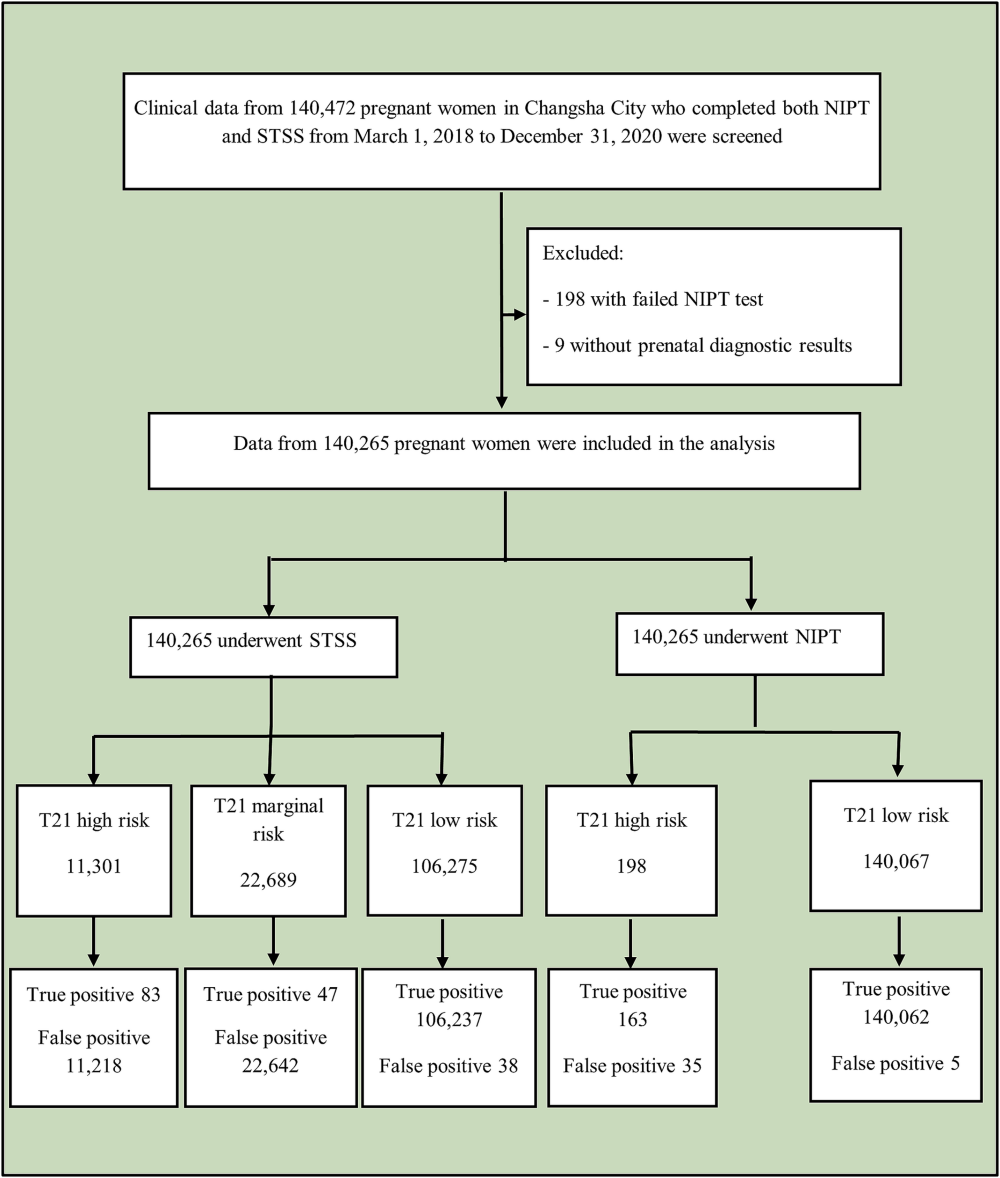
Study flowchart. NIPT, non-invasive prenatal testing; STSS, second-trimester serum screening.

This study conducted a retrospective analysis of clinical data from 140,472 pregnant women in Changsha City who underwent both NIPT and STSS between March 1, 2018, and December 31, 2020. After excluding 198 cases with failed NIPT tests and nine cases without prenatal diagnostic results, data from 140,265 women were included in the analysis (Figure 1). The data were sourced from the Changsha Public Welfare Program, organized by the Changsha Municipal Health and Health Committee and conducted by prenatal diagnosis and screening institutions across Changsha, which served as blood collection sites. These sites included Changsha Hospital for Maternal & Child Health Care, Changsha First Hospital, Changsha Central Hospital, Changsha Fourth Hospital, Changsha County Hospital for Maternal & Child Health Care, Liuyang Hospital for Maternal & Child Health Care, Ningxiang Hospital for Maternal & Child Health Care, Wangcheng District Hospital for Maternal & Child Health Care, and other hospitals. After collection, all blood samples were sent to the Changsha Hospital for Maternal & Child Health Care for centralized testing. The Changsha Hospital for Maternal & Child Health Care is responsible for implementing the city-wide NIPT project, including data aggregation, analysis, and conducting related research.

The study was approved by the Medical Research Ethics Committee of Changsha Hospital for Maternal & Child Health Care (Approval number: 2022003). This study was performed in line with the Drug Clinical Practice issued by the Chinese State Food and Drug Administration of the People’s Republic. Written informed consent was obtained from each participant prior to the start of the experiments.

### 2.3 Screening methods and cost-effectiveness analyses

#### 2.3.1 STSS

A total of 5 mL of venous blood was collected from the pregnant woman, centrifuged, and stored in a 4°C refrigerator until testing. Automated time-resolved fluorescence immunoassay was performed, incorporating combined screening with pregnancy-associated plasma protein A and free β-subunit of human chorionic gonadotropin. Fetal risk probability for trisomy 21 and trisomy 18 was calculated using the fecycle software. For trisomy 21, high-risk thresholds were set at ≥1:270 and critical risk thresholds at 1:270 to 1:1,000. For trisomy 18, high-risk thresholds were set at ≥1:350 and critical risk thresholds at 1:350 to 1:1,000.

#### 2.3.2 NIPT

A volume of 5 mL of venous blood from the pregnant woman was collected in an EDTA anticoagulant tube, stored at 4°C, and double centrifuged within 8 h. The extracted plasma containing free DNA was stored at −80°C until testing. High-throughput testing was performed using the BGISEQ500 platform (BGI Tech Solutions Co., Ltd., Shenzhen, China). Trisomy 21, 18 and 13 were determined based on sample sequencing data volume and standard sample coverage depth.

#### 2.3.3 Fetal chromosomal karyotype analysis

Amniotic fluid or umbilical cord blood was collected from pregnant women at high risk, as indicated by NIPT results. After routine cell culture, chromosomal karyotype analysis was conducted.

#### 2.3.4 Pregnancy outcome follow-up

Follow-up on pregnancy outcomes was conducted through telephone interviews and an online system with WeChat push notifications. Negative cases in NIPT were followed up through telephone interviews and online systems, with false negatives further verified using reimbursement records.

#### 2.3.5 The eight screening strategies

Based on the data, we present eight counterfactual screening strategies with the use of NIPT, STSS, and STSS Plus as first-line screening tools or a combination of these tools at different stages of the diagnostic pathway, as shown in Table 1.

**Table 1:** Summary of the Down syndrome screening strategies analyzed in this study.

Strategy 1	Universal NIPT screening	NIPT screening for all pregnant women; prenatal diagnosis for pregnant women at high risk as indicated by NIPT results.
Strategy 2	Universal STSS	STSS for all pregnant women; prenatal diagnosis for pregnant women at high risk as indicated by STSS results.
Strategy 3	Universal STSS Plus	STSS for all pregnant women; prenatal diagnosis for pregnant women at high and marginal risk as indicated by STSS results.
Strategy 4	STSS and NIPT combined screening	STSS for all pregnant women; prenatal diagnosis for pregnant women at high risk as indicated by STSS results; NIPT screening for pregnant women at marginal risk as indicated by STSS results; prenatal diagnosis for pregnant women at high risk as indicated by NIPT results.
Strategy 5	STSS and NIPT combined screening plus	STSS for all pregnant women; NIPT for pregnant women at high and marginal risk as indicated by STSS results; prenatal diagnosis for pregnant women at high risk as indicated by NIPT results.
Strategy 6	Contingent NIPT screening	NIPT screening for pregnant women aged 35 and above; STSS for pregnant women under the age of 35. Prenatal diagnosis for pregnant women at high risk as indicated by STSS results; NIPT screening for pregnant women at marginal risk as indicated by STSS results; prenatal diagnosis for pregnant women at high risk as indicated by NIPT results.
Strategy 7	Contingent NIPT screening plus	NIPT screening for pregnant women aged 35 and above; STSS for pregnant women under the age of 35; NIPT screening for pregnant women at high and marginal risk as indicated by STSS results; prenatal diagnosis for pregnant women at high risk as indicated by NIPT results.
Strategy 8	Traditional screening	Prenatal diagnosis for pregnant women aged 35 and above; STSS for pregnant women under the age of 35; prenatal diagnosis for pregnant women at high and marginal risk as indicated by STSS results.

NIPT, non-invasive prenatal testing; STSS, second-trimester serum screening.

#### 2.3.6 Cost-effectiveness analyses

Cost calculations included direct medical expenses, such as those related to NIPT, STSS, prenatal diagnosis (amniocentesis, chromosomal karyotype analysis, color Doppler ultrasound, PTC needle), and pregnancy termination. Indirect and intangible costs were not considered. Discounting was excluded from the study due to the stable and predetermined nature of the costs within the public health project. Effectiveness was measured by the number of successfully detected DS cases. The cost-effectiveness ratio was calculated to determine the cost per detected DS case. The incremental cost-effectiveness ratio was used to compare the additional cost required for extra effectiveness across different screening strategies. The safety index was assessed based on the number of normal fetal miscarriages associated with diagnosing one DS case, with a lower value indicating greater safety.


$$\mathrm{Sensitivity}=\frac{\mathrm{True}\;\mathrm{Positives}\;\left(TP\right)\;}{\mathrm{True}\;\mathrm{Positives}\;\left(TP\right)+\mathrm{False}\;\mathrm{Negatives}\;\left(FN\right)}$$

$$\begin{array}{l} \mathrm{Project}\;\mathrm{cost}=\mathrm{Number}\;\mathrm{of}\;\mathrm{NIPT}\times \mathrm{NIPT}\\ \mathrm{cost}+\mathrm{Number}\;\mathrm{of}\;\mathrm{positive}\;\mathrm{results}\times \mathrm{Prenatal}\;\mathrm{diagnostic}\\ \mathrm{fee}+\mathrm{Number}\;\mathrm{of}\;\mathrm{true}\;\mathrm{positives}\times \mathrm{Cost}\;to\;\mathrm{terminate}\;\mathrm{pregnacy} \end{array}$$

$$\mathrm{Cost}-\mathrm{effectiveness}\;\mathrm{ratio}=\frac{\mathrm{Project}\;\mathrm{cost}}{\mathrm{Number}\;\mathrm{of}\;\mathrm{true}\;\mathrm{positives}}$$

$$\begin{array}{l} \mathrm{Incremental}\;\mathrm{cost}-\mathrm{effectiveness}\;\mathrm{ratio}=\\ \frac{\left(\mathrm{Cost}\;\mathrm{of}\;\mathrm{Strategy}\;1-\mathrm{Cost}\;\mathrm{of}\;\mathrm{Strategy}\;2\right)}{\left(\mathrm{Effectiveness}\;\mathrm{of}\;\mathrm{Strategy}\;1-\mathrm{Effectiveness}\;\mathrm{of}\;\mathrm{Strategy}\;2\right)} \end{array}$$

$$\begin{array}{l} \mathrm{Accuracy}=\\ \frac{\left(\mathrm{True}\;\mathrm{positives}+\mathrm{True}\;\mathrm{negatives}\right)}{\left(\mathrm{True}\;\mathrm{positives}+\mathrm{False}\;\mathrm{positives}+\mathrm{True}\;\mathrm{negatives}+\mathrm{False}\;\mathrm{negatives}\right)} \end{array}$$

$$\begin{array}{l} \mathrm{Safety}\;\mathrm{index}=\\ \frac{\mathrm{Number}\;\mathrm{of}\;\mathrm{prenatal}\;\mathrm{diagnostic}\;\mathrm{cases}\times \mathrm{Prenatal}\;\mathrm{diagnostic}\;\mathrm{miscarriage}\;\mathrm{rate}}{\mathrm{Effectiveness}} \end{array}$$

#### 2.3.7 Univariate sensitivity analyses

A sensitivity analysis was conducted on the cost of NIPT. We focused on the incremental cost analysis, which reflects “effect” via comparisons among different scenarios. We did not conduct a probabilistic sensitivity analysis due to limitations in data availability and the resource-intensive nature of the method, which would have compromised the overall feasibility and timeliness of our study.

## 3 Results

### 3.1 Demographics

Of the 140,265 pregnant women included in the analysis, the average age was 30.15 ± 4.02 years, with the majority falling in the 25–34 age group (77.89%, 109,531/140,265). Advanced maternal age (≥35 years) accounted for 13.95% (19,612/140,265). Most of the participants (96.27%) were in their second trimester of pregnancy (14–27 weeks), with an average gestational age of 16.64 weeks. Singleton pregnancies accounted for 98.12% (137,983/140,265), and *in vitro* fertilization accounted for 4.14% (4,823/140,265) (Table 2).

**Table 2:** Participant characteristics.

Characteristic	Total population (*N* = 140,265)
Maternal age (years)	30.15 (15–53)
<25 years	11,122 (7.91%)
25–29 years	51,529 (36.64%)
30–34 years	58,002 (41.25%)
35–39 years	17,254 (12.27%)
≥40 years	2,358 (1.68%)
GA at NIPT (weeks)	16.64 (10–34)
First trimester (10–13 weeks)	4,709 (3.35%)
Second trimester (14–27 weeks)	135,386 (96.27%)
Third trimester (≥ 28 weeks)	24 (0.02%)
Unknown	146 (0.10%)
Singleton pregnancy	137,983 (98.12%)
Twin pregnancy	2,282 (1.62%)
IVF	5,823 (4.14%)

Data presented as mean (range) or n (%). GA, gestational age; IVF, *in vitro* fertilization.

### 3.2 Comparison of NIPT and STSS detection performance

The study participants underwent both NIPT and STSS, and prenatal diagnostic results were obtained. Five cases of false negatives for trisomy 21 were confirmed. The detection performance of the two methods is detailed in Table 3. NIPT demonstrated superior performance compared to STSS, identifying nearly twice as many cases of DS. NIPT consistently outperformed STSS in detecting trisomy 21 across various age groups, showing no significant differences in sensitivity and specificity. For STSS, sensitivity was notably higher in the high-risk age group compared to the low-risk group, while specificity was significantly lower in the high-risk group.

**Table 3:** The detection performance of STSS and NIPT for Down syndrome in different maternal age groups.

Description of parameters	NIPT performance			STSS performance		
	All	Maternal age < 35 years	Maternal age ≥ 35 years	All	Maternal age < 35 years	Maternal age ≥ 35 years
TP	163	100	63	83	33	50
FP	35	28	7	11,218	7,265	3,953
TN	140,062	120,522	19,540	128,879	113,285	15,594
FN	5	3	2	85	70	15
Sensitivity (95% CI)	97.02% (94.45–99.59%)	97.09% (93.84–100%)	96.92% (92.73–100%)	49.41% (41.85–56.97%)	32.04% (23.02–41.05%)	76.92% (66.68–87.17%)
Specificity (95% CI)	99.98% (99.97–99.98)	99.98% (99.97–99.99%)	99.96% (99.94–99.99%)	91.99% (91.85–92.14%)	93.97% (93.84–94.11%)	79.78% (79.21–80.34%)
PPV (95% CI)	82.32% (77.01–87.64%)	78.13% (70.96–85.29%)	90.00% (82.97–97.03%)	0.73% (0.58–0.89%)	0.45% (0.30–0.61%)	1.25% (0.91–1.59%)
NPV (95% CI)	99.99% (99.99–100%)	99.99% (99.99–100%)	99.99% (99.98–100%)	99.93% (99.92–99.95%)	99.94% (99.92–99.95%)	99.90% (99.86–99.95%)
FPR	0.03%	0.02%	0.04%	8.01%	6.03%	20.22%
FNR	2.98%	2.91%	3.08%	50.60%	67.96%	23.08%

CI, confidence interval; PPV, positive predictive value; NIPT, non-invasive prenatal testing; TP, true positives; NPV, negative predictive value; STSS, second-trimester serum screening; FP, false positives; FPR, false positive rate; TN, true negatives; FN, false negatives; FNR, false negative rate.

### 3.3 Performance and health economic analysis of different screening strategies for trisomy 21

In the Public Welfare Program in Changsha City, the cost of NIPT testing is RMB 600 per unit (21), while STSS is priced at RMB 160. The unit cost of prenatal diagnosis is RMB 2,308, including amniocentesis (RMB 800), chromosomal karyotype analysis (RMB 800), 4D color ultrasound (RMB 400), PTC needle (RMB 88), blood tests (RMB 140), and genetic counseling (RMB 80), was determined through on-site research at the hospital where the services are provided. The cost of a mid-trimester abortion is RMB 4,000 per case, with a procedure-related miscarriage risk rate of 3‰ (Table 4). Discounting was excluded from the study due to the stable and predetermined nature of the costs within the public health project. All costs were calculated in RMB based on 2018 pricing.

**Table 4:** Relative costs associated with different screening strategies for Down syndrome.

Costs, RMB	Value^a^
STSS	160
NIPT	600
Prenatal diagnosis	2,308
Amniocentesis	800
Chromosomal karyotype analysis	800
Four-dimensional color ultrasound	400
PTC needle	88
Blood tests	140
Genetic counseling	80
Mid-trimester abortion	4,000

NIPT, non-invasive prenatal testing; STSS, second-trimester serum screening.

a Data sources: the special fee schedule for Changsha Public Welfare Program of Changsha Hospital for Maternal & Child Health Care (2018).

In the comparative analysis, Strategy 1 emerged as the most effective screening method, detecting 163 true positives with only five false negatives (Table 5). It demonstrated a high accuracy rate of 99.97% and the best safety index (0.0036). Strategy 5 has the lowest cost-effectiveness ratio among the strategies analyzed in our study. For this reason, we have chosen Strategy 5 as the reference point for the computation of incremental cost-effectiveness ratio. Compared to Strategy 5, Strategy 1 detected 35 additional DS cases, with an incremental cost-effectiveness ratio of RMB 1,186,200 per additional DS case detected (Table 6). Strategy 2 was less effective, detecting 83 true positives with 85 false negatives, and lacked cost-effectiveness compared to Strategy 1. Although Strategy 3 performed better than Strategy 2, it was still inferior to Strategy 1 and resulted in a substantial number of prenatal diagnoses, leading to lower cost-effectiveness, safety, and accuracy compared to Strategies 1 and 2. Overall, this analysis suggests that universal STSS is less effective and has lower health economic value compared to universal NIPT screening. Strategies 4 and 5 demonstrated similar screening effectiveness to Strategy 3 but avoided excessive prenatal diagnoses with lower cost-effectiveness. Among these, Strategy 5 had the lowest cost-effectiveness ratio, along with higher accuracy and safety, indicating superior health economic benefits. NIPT contingent screening (Strategies 6 and 7) was more effective than STSS (Strategies 2 and 3) and combined screening (Strategies 4 and 5) but less effective than universal NIPT screening (Strategy 1). Strategy 7 detected one fewer DS case than Strategy 6 but minimized excessive prenatal diagnoses, showing significantly better cost-effectiveness, accuracy, and safety than Strategies 2, 3, 4, and 6. It performed similarly to Strategy 5 but detected six more DS cases, with the lowest incremental cost-effectiveness ratio compared to Strategy 5 (RMB 447,800 per additional DS case). Although Strategy 8 had the second-best screening outcome, detecting 142 DS cases, it resulted in the highest number of prenatal diagnoses, the highest cost-effectiveness ratio, and the highest safety index, making its safety and economic benefits the lowest among all strategies.

**Table 5:** Detection performance of different screening strategies for Down syndrome.

	Strategy 1	Strategy 2	Strategy 3	Strategy 4	Strategy 5	Strategy 6	Strategy 7	Strategy 8
True positive	163	83	130	129	128	135	134	142
False positive	35	11,218	33,860	11,245	47	7,313	63	0
True negative	140,062	128,879	106,237	128,852	140,050	132,784	140,034	140,092
False negative	5	85	38	39	40	33	34	31
Prenatal diagnosis	198	11,031	33,990	11,374	175	7,448	197	43,573
Termination of pregnancy	163	83	130	129	128	135	134	142
Sensitivity (95% CI)	97.02% (94.45–99.59%)	49.40% (41.85–56.97%)	77.38% (71.06–83.71%)	76.79% (70.40–83.17%)	76.19% (69.75–82.63%)	80.36% (74.35–86.37%)	79.76% (73.69–85.84%)	82.08% (76.37–87.80%)
Specificity (95% CI)	99.98% (99.97–99.98%)	91.99% (91.85–92.14%)	75.83% (75.61–76.06%)	91.97% (91.83–92.12%)	99.97% (99.96–99.98%)	94.78% (94.66–94.90%)	99.96% (99.94–99.97%)	100.00% (100.00–100.00%)

Prenatal diagnosis: the sum of true positives and false positives.

**Table 6:** Health economic analysis of different screening strategies for Down syndrome.

	Strategy 1	Strategy 2	Strategy 3	Strategy 4	Strategy 5	Strategy 6	Strategy 7	Strategy 8
Prenatal diagnostic cost (RMB 10,000)	45.70	2,608.27	7,844.89	2,625.12	40.39	1,719.00	45.47	10,056.65
Termination of pregnancy cost after confirmed diagnosis (RMB 10,000)	65.20	33.20	52.00	51.60	51.20	54.00	53.60	56.80
Screening cost (RMB 10,000)	8,415.90	2,244.24	2,244.24	3,605.58	4,283.64	4,106.95	4,544.83	1,930.45
Total cost (RMB 10,000)	8,526.80	4,885.71	10,141.13	6,282.30	4,375.23	5,879.95	4,643.90	12,043.90
Effectiveness (number of Down syndrome cases detected)	163	83	130	129	128	135	134	142
Cost-effectiveness ratio (RMB 10,000/case)	52.31	58.86	78.01	48.70	34.18	43.56	34.66	84.82
Incremental cost effectiveness ratio vs Strategy 5 (RMB 10,000/case)	118.62	−11.34	2,882.95	1,907.07	REF	214.96	44.78	547.76
Safety index	0.0036	0.4085	0.7844	0.2645	0.0041	0.1655	0.0044	0.9206
Accuracy	99.97%	91.94%	75.83%	91.96%	99.94%	94.76%	99.93%	99.98%

### 3.4 Univariate sensitivity analyses

**Figure 2:**
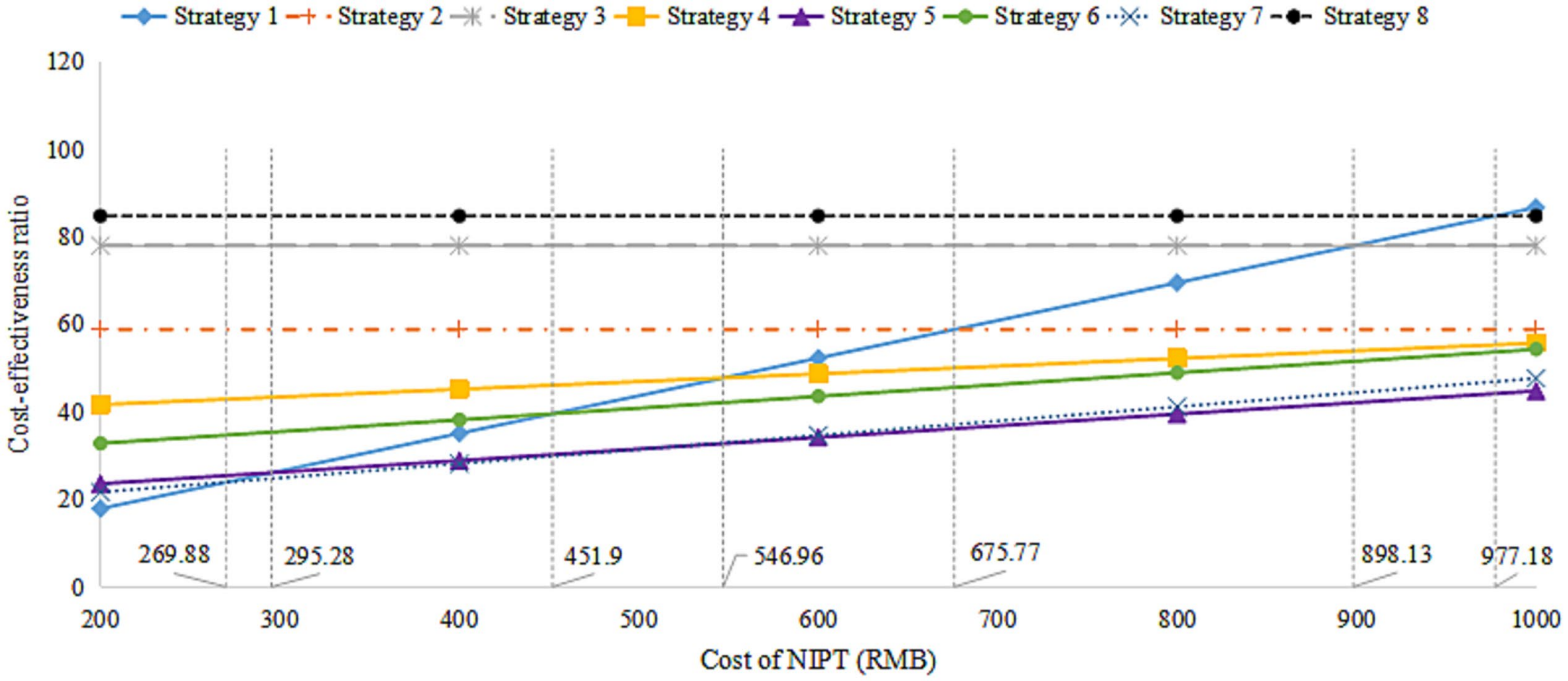
The cost-effectiveness ratio analysis for the cost of NIPT. NIPT, non-invasive prenatal testing.

We assessed the impact of varying NIPT costs on the outcomes of different screening strategies, with prices set at RMB 200, 400, 600, 800, and 1,000. As shown in Figure 2, the analysis indicated that lower NIPT costs made it more economically favorable for DS screening. Strategies 4, 6, and 5 became superior to Strategy 1 when NIPT costs exceeded RMB 546.96, 451.90, and 295.28, respectively, and were deemed “appropriate.” If the NIPT unit price fell below RMB 295.28, Strategy 1 (Universal NIPT screening) had the lowest cost-effectiveness ratio.

## 4 Discussion

In this study, NIPT showed significantly superior sensitivity, specificity, positive predictive value, and lower false positive and false negative rates compared to STSS for DS. NIPT consistently demonstrated excellent sensitivity and specificity across all age groups of pregnant women, outperforming STSS in all detection metrics. Furthermore, NIPT effectively reduced the need for invasive prenatal diagnoses, optimizing resource use and minimizing the risks associated with invasive procedures. Therefore, based on its superior detection performance, NIPT should be considered a first-tier screening method for DS.

Despite NIPT’s outstanding detection performance, its use as a primary screening method remains controversial due to high costs. Previous studies have suggested that broader use of NIPT in all pregnant women could be economically viable if the unit cost falls below $453 (22). The Public Welfare Program in Changsha City has reduced the unit cost of NIPT testing to RMB 600. Within this pricing context, Strategy 5 (STSS combined with NIPT) offers the lowest cost-effectiveness ratio per DS case detected, while Strategy 1 (universal NIPT screening) delivers the highest accuracy and safety, resulting in the most favorable screening outcomes.

In real-world health decision-making, societal economic burden is a crucial factor alongside cost-effectiveness (23). Strategy 1 can detect 35 more DS cases than Strategy 5, with an incremental cost-effectiveness ratio of RMB 1,186,200 per additional DS case detected. Thus, Strategy 1 stands out as the most effective screening option with favorable health economic benefits. Although Strategy 7 (NIPT contingent screening Plus) ranks fourth in screening effectiveness, it offers a lower cost-effectiveness ratio and incremental cost-effectiveness ratio, demonstrating excellent health economic benefits according to decision tree simulation analysis. Conversely, Strategy 8 (traditional screening methods), despite its reasonable screening effectiveness, is not recommended due to its lower safety and limited health economic value. At the current price, Strategy 1 (universal NIPT screening) was not the optimal choice compared to other strategies. Sensitivity analysis showed that reducing the price of NIPT could make universal NIPT screening (Strategy 1) a priority option. Specifically, if the NIPT price falls below RMB 295.28, Strategy 1 achieves the lowest cost-effectiveness ratio among all strategies. It is important to note that the current costs only include direct medical expenses, excluding factors such as human resource costs. Future studies are expected to show higher overall expenses once these additional costs are incorporated.

This study has several limitations. It considered only direct medical costs, excluding direct non-medical and indirect costs, which may not fully capture the societal health economic value of NIPT screening. Additionally, the analysis focused solely on STSS without comparing it to combined first-trimester serum screening and nuchal translucency ultrasound.

In conclusion, universal NIPT screening demonstrates high effectiveness, excellent detection performance, and strong safety, along with significant health economic benefits. As the cost of NIPT decreases, it is expected to become the most effective and economically advantageous screening strategy.

## Data Availability

The original contributions presented in the study are included in the article/supplemental material, further inquiries can be directed to the corresponding authors.
